# Triacylglycerol Composition of Seed Oil from *Corema album* Berries

**DOI:** 10.3390/molecules30040914

**Published:** 2025-02-16

**Authors:** Carmen Martín-Cordero, Enrique Martinez-Force, Nuria Acero de Mesa, Dolores Muñoz-Mingarro, Antonio J. León-González

**Affiliations:** 1 Department of Pharmacology, Faculty of Pharmacy, University of Seville, C/P. García González, 2, 41012 Seville, Spain; ajleon@us.es; 2 Department of Biochemistry and Molecular Biology of Plant Products, Instituto de la Grasa-CSIC, Ctra. de Utrera km 1, 41013 Seville, Spain; emforce@ig.csic.es; 3 Pharmaceutical and Health Science Department, Pharmacy Faculty, San Pablo-CEU University, Campus Monteprincipe, 28668 Boadilla del Monte, Madrid, Spain; nacemes@ceu.es; 4 Chemistry and Biochemistry Department, Pharmacy Faculty, San Pablo-CEU University, Campus Montepríncipe, 28668 Boadilla del Monte, Madrid, Spain; dmumin@ceu.es

**Keywords:** *Corema album* seed oil, fatty acid composition, triacylglycerols, fatty acid distribution, tocopherols, tocotrienols, tocochromanols, melting profile, atherogenicity index, thrombogenicity index

## Abstract

The seeds of *Corema album* are considered a by-product in fruit processing. This study aimed to determine the oil contents in seeds and characterize their triacylglycerol contents through a comparative analysis using three extraction solvent systems: hexane (Soxhlet method), hexane–isopropanol (Hara–Radin method), and methanol–chloroform–water (Bligh–Dyer method). The extracts were analyzed by gas chromatography/mass spectrometry and HPLC. The composition of fatty acids and triacylglycerols was determined, as were the allocation of fatty acids across the *sn*-2 and *sn*-1,3 positions, tocopherol and tocotrienol profile, and melting behavior through differential scanning calorimetry. Furthermore, the atherogenicity (IA) and thrombogenicity (IT) cardiovascular health indices were also calculated. The oil predominantly contained unsaturated fatty acids, and α-linolenic acid made up 45.8% of the total, along with a reduced n-6/n-3 fatty acid ratio (0.75). The α-linolenoyl chain primarily occupied the *sn*-1,3 (45.9%) and *sn*-2 (39.1%) positions. γ-tocotrienol was the most abundant tocochromanol. The melting curve of oil suggests the presence of fractions with a low melting point, composed of triacylglycerols containing polyunsaturated fatty acids. The oil exhibits low values for IA and IT of 0.05 and 0.04, respectively. *Corema* seed oil has potential health benefits thanks to its rich composition in the essential fatty acid, α-linolenic acid, the low proportion of n-6/n-3 fatty acids, and the low values of IA and IT.

## 1. Introduction

*Corema album* (L.) D. Don, commonly known as camariña or white crowberry, is an evergreen belonging to the Ericaceae family, endemic to the southern Atlantic coast of Europe. It thrives in sandy coastal regions, extending from northern Galicia to Tarifa in the south [1]. Archaeological evidence suggests that its fruits were part of the diet of prehistoric human populations in the Iberian Peninsula [2]. The berries measure between 5 and 10 mm in diameter, are white or pale pink with an acidic flavor, and contain three large seeds inside, which measure 3.75 mm in length and 2.81 mm in width [3,4]. Traditionally, the fruit is consumed fresh and also employed to make drinks, such as liquors and lemonades, as well as jellies [5]. This fruit, in the form of lemonade, has been used in folk medicine for the treatment of fever and intestinal parasites [6]. In previous phytochemical studies, we have highlighted the presence of phenolic compounds in the juice, such as phenolic acids, flavonols, flavanones, flavanol, and a coumarin [7,8,9]. Moreover, the juice exhibits antioxidant and neuroprotective properties [9,10].

Subsequent to the production of berry juice, the remaining puree contains seeds and peel. The seeds can be separated from the puree and subsequently utilized to extract oil [11].

Berry seed oils, such as those of red raspberries, strawberries, grapes, cranberries, crowberries, raspberries, and blueberries, contain triacylglycerols (TAGs) rich in two types of essential polyunsaturated fatty acids (PUFAs): omega-6 (n-6) and omega-3 (n-3). These PUFAs cannot be biosynthesized by the body and must be obtained through the diet [11,12].

Currently, Western diets are rich in n-6 polyunsaturated fatty acids (PUFAs) and have a very high n-6/n-3 ratio, which contributes to the development of various cardiovascular, inflammatory, and autoimmune diseases, and even cancer. However, diets rich in n-3 PUFAs (with a low n-6/n-3 ratio) exert protective effects [13,14]. In particular, the essential fatty acid known as alpha-linolenic acid (18:3, ω-3, n-3, ALA) offers numerous health benefits, including reduction in the risk of coronary heart disease, strokes, inflammation, and certain cancers. It also plays a crucial role in the development and function of the brain and retina [13,15].

The molecular structure of TAGs is crucial for the improvement in their digestibility, small intestine absorption, and bioavailability and is therefore also crucial for health, with FAs at the sn-2 position being easily absorbed [16].

Tocopherol and tocotrienol isomers are generally analyzed in vegetable oils as the main antioxidant constituents. They are important for both the oxidative stability and nutritional quality of edible oils [17]

The nutritional value of seed oils is often determined by different health indices, such as the n6/n3 ratio, the lipid quality index, the atherogenicity index, and the thrombogenicity index, which reflect the impact on cardiovascular health [18].

The aim of this study was to determine the oil contents in the seeds and to analyze their triacylglycerol composition by gas chromatography/mass spectrometry. A comparative study was conducted using three extraction solvent systems. We determined the composition of fatty acids, the triacylglycerols, the allocation of fatty acids across the *sn*-2 and *sn*-1,3 positions, and the melting behavior by means of differential scanning calorimetry. Furthermore, the n6/n3 ratio, the lipid quality index, and the atherogenicity (IA) and thrombogenicity (IT) cardiovascular health indices were also calculated.

## 2. Results and Discussion

### 2.1. Oil Content

The oil of *Corema* seeds was obtained using three different extraction methods: first, hot extraction with a Soxhlet extractor (H); and then either the Hara and Radin method (HR) or Bligh and Dyer method (BD) with sonication. Solvents of varying polarities were used: hexane (H), hexane–isopropanol (HR), and methanol–chloroform–water (BD). The oil contents (% DW) are presented in Table 1. This study reveals only minor differences between the samples. Depending on the extraction method, the oil content ranges from 2.77% (H) to 2.1% (BD). These values are considerably lower than those reported in the literature for oil contents in other berry seeds, which range from 30.6% for small cranberry seeds (*Vaccinium oxycoccos* L.) to 4.7% for bearberry seeds (*Arctostaphylos uva-ursi* (L.) Sprengel), both of which belong to the Ericaceae family [12].

### 2.2. Composition of Fatty Acids

The percentage contents of fatty acids from particular groups of saturated (SFA), monounsaturated (MUFA), and polyunsaturated fatty acids (PUFA) and the ratio of n-3 to n-6 fatty acids are summarized in Table 2. The analysis reveals that the predominant fatty acids are α-linolenic (ALA) and linoleic (LA) acids. ALA is the most abundant fatty acid in all extracts, which accounts for 44.99–47.12% of the total fatty acids, followed by LA, which ranges from 32.54% to 35.64%. The extracts also contain monounsaturated fatty acids, with oleic acid (OA) the most prominent (11.66–13.04%) and cis-vaccenic acid present in only trace amounts (0.32–0.48%). Additionally, *Corema* seed oil contains small amounts of saturated fatty acids, such as palmitic and stearic acids at 5% and 2%, respectively.

Berry seed oils, including those from grapeseed (*Vitis vinifera* L.), red raspberry (*Rubus idaeus* L.), blackberry (*Rubus fruticosus* L.), blueberry (*Vaccinium corymbosum* L.), strawberry (*Fragaria × ananassa* (Duchesne ex Weston) Duchesne ex Rozier), and cranberry (*Vaccinium macrocarpon* Aiton) constitute a rich source of polyunsaturated fatty acids, with LA the predominant component in all thereof [16,18,19].

Our results are consistent with those found in oils from wild berry seeds such as lingonberry (*Vaccinium vitis-idaea* L.), blueberry (*Vaccinium myrtillus* L.), whortleberry (*Vaccinium uliginosum* L.), small cranberry (*Vaccinium oxycoccos* L.), bearberry (*Arctostaphylos uva-ursi* (L.) Spreng.), black bearberry (*Arctous alpina* (L.) Nied. Spreng.), Southern crowberry (*Empetrum nigrum* L.), and Northern crowberry (*Empetrum nigrum* subsp. *hermaphroditum* (Hagerup) Böcher), which all belong to the Ericaceae and Empetraceae families, both of which are within the order Ericanae, where the predominant fatty acids are ALA (35.1–49.7%), LA (31.3–40.7%), and OA (12.5–21.6%) [12,20].

ALA is an essential fatty acid that must be ingested through the diet via edible oils rich in this component, such as flaxseed, perilla seed, chia seed, and rapeseed. ALA is metabolized to eicosapentaenoic acid (EPA) and docosahexaenoic acid (DHA) by desaturases and elongases in humans. According to various studies, ALA has an anti-metabolic syndrome, is anti-cancer, anti-inflammatory, antioxidant, and anti-obesity, and provides neuroprotection and regulation of the intestinal flora properties [21].

This study highlights for the first time that *Corema* seed oil can be a good source of essential fatty acids (EFAs) in the diet, primarily in the form of polyunsaturated fatty acids (PUFAs), and more specifically, ALA.

Furthermore, in Western countries, the increasing incidence of diseases, such as diabetes, rheumatoid arthritis, inflammatory bowel disease, obesity, asthma, cancer, and depression, has been associated with a higher ratio of n-6 to n-3 fatty acids in the diet. Excessive consumption of n-6 fatty acids promotes the biosynthesis of pro-inflammatory eicosanoids, which are implicated in an inappropriate inflammatory response of the immune system. In contrast, the biosynthesis of anti-inflammatory eicosanoids, formed from n-3 fatty acids in the metabolic pathway, plays a crucial role in regulating the inflammatory response and in modulating the immune system, helping to prevent chronic inflammatory diseases, such as arthritis and cardiovascular diseases [22,23]. To have a positive impact on health, it has been shown that the optimal ratio of n-6 to n-3 fatty acids should lie between 1:1 and 4:1 [23]. *Corema* seed oil has a beneficial n-6 to n-3 fatty acid ratio, 0.75 (1:0.75), which could help reduce the risk of chronic inflammatory conditions, cardiovascular diseases, and other disorders associated with inflammation. Furthermore, this oil is characterized by low atherogenicity and thrombogenicity index values (0.05 and 0.04, respectively), even lower than those found in berry seed oils such as cranberry, blackcurrant, redcurrant, strawberry, and chokeberry, which are considered important components of dietary prevention and treatment for cardiovascular diseases [24].

### 2.3. TAG Composition

The analysis of the composition of TAGs in *Corema* seed oil showed a wide variability, which was very similar in all cases. Twenty molecular species of TAGs were identified and quantified (Table 3). Among all of the TAGs, LLLn + OLLn (21.74 ± 1.31 g/100 g), LLnLn (17.21 ± 1.38 g/100 g), LLL + OLLn (15.86 ± 1.55 g/100 g), and LnLnLn (8.9 ± 0.84 g/100 g) were the predominant molecular species in the three methods of extraction employed. Other TAG species, such as OLL + OOLn, PLLn, and PLL, were present in low quantities, with their percentages scarcely surpassing 8% of the total TAG species.

The analysis of TAGs in an oil is of considerable interest since their composition can significantly affect the physical, chemical, and nutritional properties of the oil, as well as its oxidative stability and thermal behavior. The abundance of TAGs with unsaturated fatty acids, such as LA and ALA, indicates that the oil has a low melting point and is more susceptible to oxidation. From a nutritional perspective, this abundance has a favorable impact on health, since both are essential fatty acids, meaning that the human body cannot biosynthesize them and they must be obtained through the diet. These acids have anti-inflammatory and lipid-lowering effects and support human cardiovascular and autoimmune systems. They exert a positive effect on the intestinal microflora and the skin [25].

### 2.4. Fatty Acid Distribution Between sn-2 and sn-1,3 Positions of Triacylglycerols

The different classes of the lipid compounds are separated on silica plates subsequent to lipase digestion. Fatty acids are separated from triacylglycerols (TAGs), diglycerides (DAGs), and monoglycerides (MAGs). The selectivity of the lipase employed removes fatty acids from the *sn*-1 and *sn*-3 positions of triacylglycerol, leaving the MAGs with only the fatty acid at the *sn*-2 position. This step is crucial for the determination of the suitability of the oil for valorization, since the organism primarily utilizes the MAGs at the *sn*-2 position. Ingested TAGs are rapidly degraded to DAGs and then to MAGs. The percentage of MAGs is determined using the following equation, with the quantification of TAGs and DAGs.

FA (*sn*-2) = 3 × FA(Sn1,2,3) − 2 × FA(Sn1,3)

The results are shown in Table 4, whereby 18:2 and 18:3 are mostly found in the *sn*-2 central position of the TAGs. This result is promising since these two fatty acids are essential to the organism. The oil could be revalorized as a food complement or in alimentary industries.

From a nutritional perspective, in addition to the fatty acid composition of triacylglycerols, the distribution of these fatty acids between the internal and external positions in the triacylglycerol is crucial, since they are not equivalent. The majority of fatty acids in food are contained in triacylglycerols (TAGs), which are preferentially absorbed in the duodenum as *sn*-2 monoglycerides and exhibit higher bioavailability, and thus have a greater impact on health [26].

### 2.5. Composition of Tocochromanols

**Figure 1:**
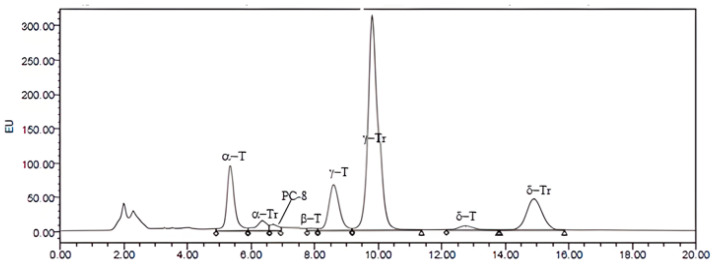
HPLC chromatogram of *Corema* seed oil obtained by Soxhlet with hexane. T, tocopherol; Tr, tocotrienol. PC-8, Plastochromanol (25 µg/mL) was added to the standard solutions as an antioxidant.

Data presented in Figure 1 and Table 5 show that *Corema* seed oil contains four forms of tocopherols, α, β, γ, and δ, and three forms of tocotrienols, α, β, and γ.

This analysis reveals that, in *Corema* seed oil, tocotrienols predominate (72.47%) and γ-tocotrienol constitutes 57.51% of the tocochromanols. The higher susceptibility of *Corema* oil to oxidation, due to its high content of polyunsaturated fatty acids (PUFAs), results in a shorter storage time, since oxidation negatively affects both its sensory properties. Tocotrienols and tocopherols serve as powerful lipid-soluble antioxidants in oils and enhance their oxidative stability [27].

Our results agree with those obtained for cranberry seed oil, which is the richest known source of natural γ-tocotrienol. Vaccinium macrocarpon Aiton is a species phylogenetically very close to *Corema album*, both of which belong to the Ericaceae family [28].

### 2.6. Differential Scanning Calorimetry (DSC) of Corema Seed Oil

**Figure 2:**
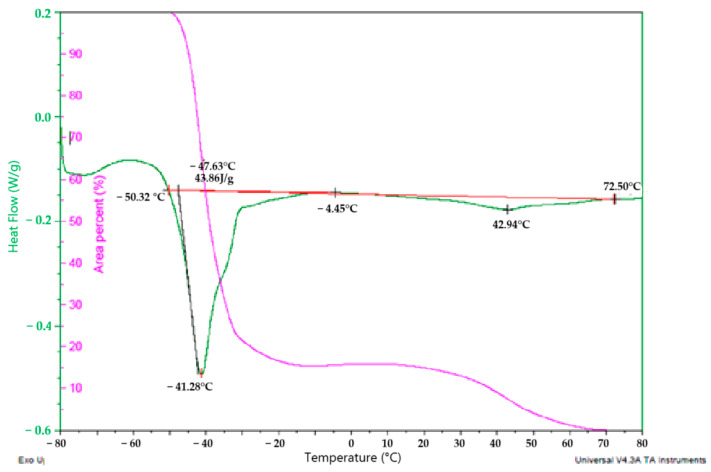
DSC curve recorder for *Corema* seed oil.

The DSC curve of the heating of *Corema* seed oil from −80 °C to 80 °C at a rate of 5 °C/min is presented in Figure 2. A shoulder between −70 and −50 °C corresponds to the rearrangement of the crystallization from the lipids just before melting. It can be observed that *Corema* seed oil crystallizes at a lower temperature.

An endothermic peak appears at −41.28 °C due to the occurrence of low-melting fractions of triacylglycerols (TAGs), which is typical for TAGs with high polyunsaturated fatty acids [29]. This is consistent with the findings in Table 2, which demonstrate a high proportion of polyunsaturated fatty acids (PUFAs) in *Corema* seed oil. Similar shapes of DSC curves are obtained for strawberry, blackberry, and raspberry seed oils, in which an endothermic peak is observed at −40.23 °C, −40.35 °C, and −40.01 °C, respectively [22,26]. Furthermore, a second small shoulder between −35 and −30 °C corresponds to the melting of DAG and MAG esters. Additionally, a second small endothermic peak is observed at approximately 43 °C, which appears to correspond to the melting point of saturated fractions of other unsaponifiable compounds, such as waxes and hydrocarbons. The melting enthalpy (∆Hm) of the endothermic thermodynamic reaction is 43.85 J/g. This value is in accordance with the melting enthalpies of blackberry and raspberry seed oils, at 72.44 °C and 63.95 °C, respectively [30].

The pink curve shows the solid/liquid proportions while the temperature increases. At room temperature (20–25 °C), approximately 85% of the oil is in the liquid phase and 15% is in the solid phase. This characteristic can be of interest in the formulation of margarines and their use in coatings, since it results in a smoother texture and a more fluid coverage [31]. Furthermore, it can enhance the nutritional properties of margarines due to its low content of saturated fatty acids and high content of ALA.

## 3. Materials and Methods

### 3.1. Plant Material

Wild ripe *Corema album* berries (1 kg) were harvested by hand from 12 independent bushes in September 2022, in Doñana National Park (Huelva, SW Spain) 37°04′10.15″ N–6°41′15.41′ W, and identified by Dr. Mari Cruz Diaz Barradas, from the Department of Plant Biology and Ecology, University of Seville. The berries were pooled and cleaned in order to remove damaged fruit and leaves, and then stored in polyethylene bags at −40 °C until analysis. The berries were pressed to obtain the juice, and the seeds were subsequently removed and air dried at room temperature.

### 3.2. Chemicals

Hexane, methanol, chloroform, isopropanol, toluene, sulfuric acid, and heptane were obtained from Panreac Química (Barcelona, Spain). Methyl ester (FAME) standards were acquired from Nu-Chek-Prep (Elysian, MN, USA). Tocopherol standards were provided by Sigma–Aldrich (Saint-Quentin Fallavier, France).

### 3.3. Oil Extraction

The dried seeds were ground into powder using a Culatti SDFH 48 grinder and extracted by three different methods using solvents of varying polarity, in order to compare whether one method was better than another. First, the oil was extracted using a Soxhlet apparatus with hexane (H) as a non-polar solvent for 15 h. The Hara and Radin (HR) method was then used, with a hexane–isopropanol mixture in a 3:2 *v*/*v* ratio by sonication for 15 min [32], and, finally, the Bligh and Dyer (BD) method was employed, using a methanol–chloroform–water mixture (2:1:0.8 *v*/*v*), also by sonication for 15 min [33]. To quantify the total oil content, the extracts were weighed, and the results were expressed as a percentage of the dry seed weight. The analyses were carried out in triplicate.

### 3.4. Fatty Acid Composition

The lipid fractions obtained by the three methods were analyzed by dissolving 6 mg of oil in 500 μL of chloroform. The solution was then fractionated on a Li-Chrolut silica gel cartridge (40–63 μm, Merck) under vacuum, followed by equilibration with 2 mL of chloroform [34]. The total lipid extract was loaded onto the column, which was subsequently washed with 15 mL of chloroform to elute neutral lipids. The polar lipids were quantitatively recovered by washing the column with 10 mL of methanol. The fatty acid composition was determined by esterifying 6 mg of the oil fractions into fatty acid methyl esters (FAMEs) using a methanol/toluene/sulfuric acid solution (85/15/2.5 *v*/*v*/*v*) for 1 h at 80 °C [35]. The FAMEs were then extracted with 2 mL of heptane and analyzed by gas chromatography (GC) on an Agilent 6890 system (Palo Alto, CA, USA). The column used was a Supelco SP-2380 fused silica capillary column (30 m, 0.25 mm inner diameter, 0.20 μm film thickness; Bellefonte, PA, USA), with hydrogen as the carrier gas at 28 cm/s. The oven and detector temperatures were set at 170 °C and 200 °C, respectively. The identification of the different methyl esters was based on the comparison of their retention times with known methyl ester (FAME) standards. The nutritional evaluation of the fatty acid composition was performed by calculating the atherogenicity index (AI), thrombogenicity index (TI) according to Ulbricht and Southgate (1991) [36], and the polyunsaturated-to-saturated fatty acid ratio (PUFA/SFA).

### 3.5. TAG Analysis

The analysis of the triacylglycerol (TAG) contents in the different oil fractions was conducted by injecting 1 μL aliquots of a 2.5 mg/mL oil solution in 2 mL of heptane into an Agilent 7890 gas chromatograph (Palo Alto, CA) [34]. The chromatographic setup was similar to that for methyl esters but included a Quadrex Aluminum-Clad 400-65HT column (30 m length, 0.25 mm inner diameter, 0.1 μm film thickness; Woodbridge, CT, USA) and a flame ionization detector. The injector and detector were set to 370 °C and 360 °C, respectively, with hydrogen as the carrier gas at a linear velocity of 50 cm/s and a split ratio of 1:80. The oven was maintained at 335 °C, and TAGs were eluted by applying a head pressure gradient from 100 to 180 kPa. TAG species were identified by comparing their retention times with those stored in our database from previous analyses of oils with known TAG compositions.

### 3.6. TAG Lipolysis

For the positional analysis of sn-2 fatty acids in TAGs, 7.6 mg of purified TAGs was hydrolyzed with 3 mg of pancreatic lipase in 1 mL of 1 M Tris-HCl buffer (pH 8), 0.1 mL of 22% CaCl_2_, and 0.25 mL of 0.1% deoxycholate. The reaction was stopped after approximately 60% hydrolysis of the TAGs (1–2 min) by adding 0.5 mL of 6 N HCl. The lipids were extracted three times with 2.5 mL aliquots of ethyl ether, and the reaction products were separated via thin-layer chromatography (TLC). Bands corresponding to free fatty acids and sn-2-monoacylglycerol, representing the sn-1,3 and sn-2 positions of the TAGs, were scraped from the TLC plate, trans-methylated, and analyzed by GC. The procedure’s validity was confirmed by comparing the fatty acid composition of the original TAGs with that of the remaining TAGs after partial hydrolysis [37].

### 3.7. Analysis of Tocochromanols

*Corema* seed oil (10 mg) obtained by Soxhlet extraction with hexane was dissolved in 1 mL of hexane. The oil was analyzed using normal-phase, high-performance liquid chromatography (HPLC). The chromatographic system used was a Merck Supersphere Si60 column (particle size 4 μm, 250 × 64 mm), with elution using a mobile phase of hexane: 2-propanol (99:1) at a flow rate of 1 mL/min. A fluorescence detector (Shimadzu RF535, Kyoto, Japan) was employed, with an excitation wavelength set at 290 nm and an emission wavelength at 330 nm. The injection volume of the sample was 20 µL. The relative quantification of compounds was performed by integrating the area under the curve of the chromatographic peaks.

### 3.8. Calorimetric Analysis by DSC

The melting and crystallization profiles of the oils were evaluated using differential scanning calorimetry (DSC) on a Q2000 calorimeter (TA Instruments, New Castle, DE, USA), calibrated with indium. Samples of approximately 7 mg of melted oil were transferred to aluminum pans, and the weight of the pans and the samples was precisely measured using a Sartorius M2P electronic microbalance (Sartorius AG, Göttingen, Germany). Melting intervals were determined by heating the oils to 90 °C for 5 min, followed by a cooling cycle to −40 °C at 20 °C/min, with a temperature modulation amplitude of ±0.8 °C every 60 s. The temperature was then ramped back to 90 °C at a rate of 5 °C/min. Modulated DSC (MDSC) could potentially be utilized to investigate the thermal transitions of the oil blends, allowing for the separation of overlapping phenomena such as melting/recrystallization and other subtle phase transitions, maintaining the resolution [38,39]. By using MDSC, it is possible to distinguish between reversible and non-reversible thermal events [40]. In this study, only the reversible heat flow signal was considered to analyze the melting behavior of the oils.

The solid fat content (SFC) was calculated by continuous integration of the DSC melting profiles using the TA Universal Analysis software V4.3A. Additionally, the crystallization process was tracked by completely melting the oils at 90 °C for 5 min and collecting the heat flow data as the temperature decreased to −70 °C at 10 °C/min. The data integration and analysis were carried out using the TA Universal Analysis program, which excluded any irreversible transitions such as polymorphic transformations that may have occurred during the melting process.

### 3.9. Statistical Analysis

The mean and standard deviation in the three determinations performed in each extraction method were given.

## 4. Conclusions

Our results suggest that *Corema* seed oil may serve as an excellent dietary source for ALA and LA and may be included in the human diet, as a gourmet oil or nutraceutical oil for the improvement of the n-6/n-3 ratio. The major bioavailability of ALA could have a high health effects, such as anti-metabolic syndrome; anti-cancer, anti-inflammatory, antioxidant, and anti-obesity effects; provision of neuroprotection; and regulation of the intestinal flora properties. Further study of the oxidative stability of this new PUFA-rich oil is necessary, and analysis of the unsaponifiable fraction is therefore essential, primarily focusing on the content of sterols, and on that of phenolic compounds due to their antioxidant activity. Lastly, it would be advantageous for future research to focus on obtaining *Corema* seed oil through cold-press extraction, since this oil remains liquid at room temperature, which facilitates the extraction process. In addition to conducting a comprehensive quantitative analysis of its components, this extraction method is ideal for edible oils that serve as functional foods or nutraceuticals. Moreover, cold press extraction offers several benefits over conventional methods, including greater safety, superior quality, enhanced energy efficiency, and a lower environmental impact. The oil obtained through this method will exhibit greater oxidative stability, reduced nutrient loss, and improved organoleptic properties.

## Figures and Tables

**Table 1:** Oil contents (% DW) of *Corema* seeds from three methods using three system solvents.

	H	HR	BD
% Oil	2.77 ± 0.40	2.06 ± 0.19	2.17 ± 0.19

(H) hexane, (HR) hexane/isopropanol, and (BD) methanol–chloroform–water.

**Table 2:** FA composition (percentage of total FA) using three system solvents of extraction of oil seed: (H) hexane, (HR) hexane/isopropanol, and (BD) methanol–chloroform–water.

FA	H	HR	BD	H–HR–BD
16:0	5.00	4.77	5.07	4.95 ± 0.16
18:0	1.80	2.15	2.22	2.06 ± 0.23
18:1 (9)	13.04	12.08	11.66	12.26 ± 0.71
18:1 (11)	0.48	0.36	0.32	0.39 ± 0.08
18:2 (n-6)	32.54	35.64	34.51	34.23 ± 1.57
18:3 (n-3)	47.12	44.99	45.54	45.88 ± 1.11
18:2 (n-6)/18:3 (n-3)	0.69	0.79	0.76	0.75 ± 0.05
SFA	6.80	6.93	7.29	7.01 ± 0.25
MUFA	13.52	12.44	11.98	12.65 ± 0.79
PUFA	79.65	80.63	80.05	80.11 ± 0.49
PUFA/SFA	11.71	11.63	10.98	11.43 ± 1.94
AI	0.05	0.05	0.05	0.05
TI	0.04	0.04	0.04	0.04

Notes: Results are given as means ± SD from three methods. The FAs are 16:0, palmitic acid; 18:0, stearic acid; 18:1 (9), oleic acid; 18:1 (11), cis-vaccenic acid; 18:2 (n6), linoleic acid; 18:3 (n3), α-linolenic acid. SFA, saturated, MUFA, monounsaturated, and PUFA, polyunsaturated fatty acids; AI, atherogenicity index; and TI, thrombogenicity index.

**Table 3:** TAG profiles (percentage of the total TAGs) of *Corema* seed oil in terms of the three methods of extraction with different polarities.

TAG Type	H	HR	BD	H–HR–BD
POP	0.22	0.22	0.30	0.25 ± 0.05
PLP	0.30	0.22	0.30	0.62 ± 0.05
PLnP	0.27	0.22	0.30	0.26 ± 0.04
POS	0.18	0.22	0.29	0.23 ± 0.06
POO	0.57	0.22	0.29	0.36 ± 0.19
PSL	0.26	0.48	0.66	0.47 ± 0.20
POL	1.98	2.36	2.16	2.17 ± 0.19
PLL + POLn	4.78	5.75	4.84	5.12 ± 0.54
PLLn + PLnLn	6.00	6.63	5.22	5.95 ± 0.71
SOO + SLS + SLnL	2.12	3.61	3.48	3.07 ± 0.83
OOO	1.28	1.72	1.76	1.59 ± 0.27
SOL	0.84	1.49	0.00	0.78 ± 0.75
OOL + SOLn	2.48	2.92	2.74	2.71 ± 0.22
SLL	1.30	1.91	1.15	1.45 ± 0.40
OLL + OOLn	7.66	8.19	6.97	7.61 ± 0.61
SLLn + SLnLn	1.91	2.05	1.74	1.9 ± 0.16
LLL + OLLn	17.18	16.24	14.15	15.86 ± 1.55
LLLn + OLnLn	23.15	21.52	20.55	21.74 ± 1.31
LLnLn	18.7	15.96	16.98	17.21 ± 1.38
LnLnLn	8.82	8.1	9.78	8.9 ± 0.84

Notes: Results are given as means ± SD from three methods of extraction. Values are calculated as the percentage of the total. The order of the fatty acids in the TAG type does not indicate the TAG structure (PEL = PLE = LPE). P, palmitic acid (16:0); S, stearic acid (18:0); O, oleic acid (18:1); L, linoleic acid (18:2); Ln, α-linolenic (18:3). PLL + POLn, PLLn + PLnLn, SOO + SLS + SLnL, OOL + SOLn, OLL + OOLn, SLLn + SLnLn, LLL + OLLn, and LLLn + OLnLn are not separated, and the value represents the sum of the species in oil with these TAG species.

**Table 4:** Distribution of FA between the *sn*-2 and *sn*-1,3 positions in TAG molecules.

FA	FA(Sn2) MAG %	FA (Sn1, 2, 3) TAG %	FA (Sn1, 3) DAG %
16:0	3.31	9.49	12.58
18:0	5.76	4.99	4.61
18:1 (9)	16.64	11.11	8.35
18:1 (11)	0	0.6	0.9
18:2	35.18	30.13	27.61
18:3	39.10	43.67	45.96

16:0, palmitic acid; 18:0, stearic acid; 18:1 (9), oleic acid; 18:1 (11), cis-vaccenic acid; 18:2 (n-6), linoleic acid; 18:3 (n-3), α-linolenic acid.

**Table 5:** Percentage composition of tocochromanols from *Corema* seed oil obtained by the Soxhlet method with hexane.

Tocopherols				Tocotrienols			
26.3%				72.47%			
γ + δ		α + β		γ + δ		α + β	
13.59%		12.71%		69.73%		2.74%	
α	β	γ	δ	α	β	γ	δ
12.19%	0.52%	11.93%	1.66%	2.74%	-	57.51%	12.22%

## Data Availability

The original contributions presented in this study are included in the article.

## Abbreviations

The following abbreviations are used in this manuscript:

IA	atherogenicity index
IT	thrombogenicity index
H	Soxhlet extraction with hexane
HR	Hara and Radin method
BD	Bligh and Dyer method
DW	dry weight
TAGs	triacylglycerols
PUFA	polyunsaturated fatty acid
MUFA	monounsaturated fatty acid
SFA	saturated fatty acid
n-3	omega-3
n-6	omega-6
ALA	α-linolenic acid
LA	linoleic acid
EPA	eicosapentaenoic acid
DHA	docosahexaenoic acid
POP	dipalmitoyl–oleoyl–glycerol
PLP	dipalmitoyl–linoleoyl–glycerol
PLnP	dipalmitoyl–linolenoyl–glycerol
POS	palmitoyl–oleoyl–stearoyl–glycerol
POO	dioleoyl–palmitoyl–glycerol
PSL	palmitoyl–stearoyl–linoleoyl–glycerol
POL	palmitoyl–oleoyl–linoleoyl–glycerol
PLL	dilinoleoyl–palmitoyl–glycerol
POLn	palmitoyl–oleoyl–linolenoyl–glycerol
PLLn	palmitoyl–linoleoyl–linolenoyl–glycerol
PLnLn	dilinolenoyl–palmitoyl–glycerol
SOO	dioleoyl–stearoyl–glycerol
SLS	distearoyl–linoleoyl–glycerol
OOO	triolein
SOL	stearoyl–oleoyl–linoleoyl–glycerol
OOL	dioleoyl–linoleoyl–glycerol
SOLn	stearoyl–oleoyl–linolenoyl–glycerol
SLL	dilinoleoyl–stearoyl–glycerol
OLL	dilinoleoyl–oleoyl–glycerol
OOLn	dioleoyl–linolenoyl–glycerol
SLLn	stearoyl–linoleoyl–linolenoyl–glycerol
SLnLn,	dilinolenoyl–stearoyl–glycerol
LLL	trilinolein
OLLn	oleoyl–linoleoyl–linolenoyl–glycerol
LLLn	dilinoleoyl–linolenoyl–glycerol
OLnLn	dilinolenoyl–oleoyl–glycerol
LLnLn	dilinolenoyl–linoleoyl–glycerol
